# Supplementary material for: Shared Treg dysregulation underlies psoriasis vulgaris and atopic dermatitis through the PHF19-PRC2 epigenetic axis
Source: Front Immunol. 2026 Aug 20;17:1932788. doi: 10.3389/fimmu.2026.1932788 (PMC13538097; doi:10.3389/fimmu.2026.1932788)
Supplement: Supplementary file 3 [file DataSheet3.zip › Additional files/Additional file 6/02.RABEP1.forest.pdf]

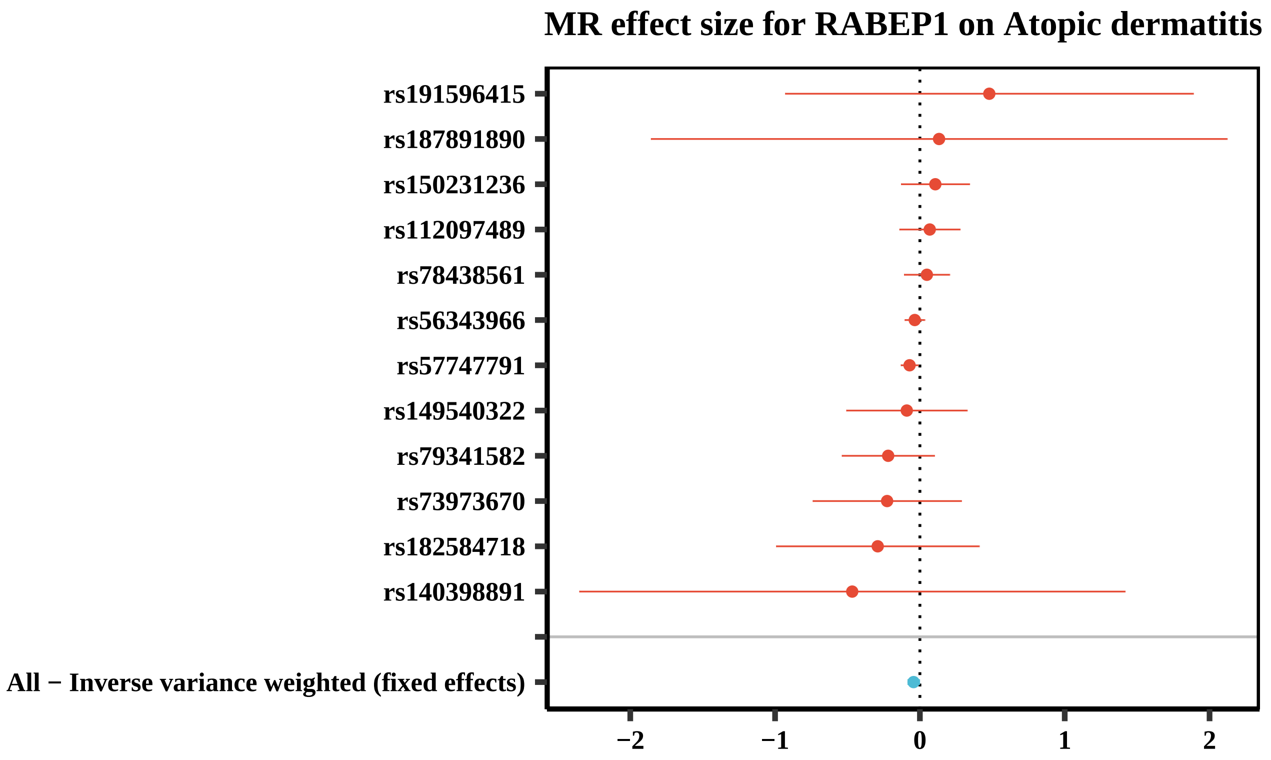

MR effect size for RABEP1 on Atopic dermatitis
rs191596415
rs187891890
rs150231236
rs112097489
rs78438561
rs56343966
rs57747791
rs149540322
rs79341582
rs73973670
rs182584718
rs140398891
All − Inverse variance weighted (fixed effects)
−2
−1
0
1
2
